# Studies on Bioflocculant Production by *Arthrobacter* sp. Raats, a Freshwater Bacteria Isolated from Tyume River, South Africa

**DOI:** 10.3390/ijms13011054

**Published:** 2012-01-19

**Authors:** Leonard V. Mabinya, Sekelwa Cosa, Uchechukwu Nwodo, Anthony I. Okoh

**Affiliations:** Applied and Environmental Microbiology Research Group (AEMREG), Department of Biochemistry and Microbiology, University of Fort Hare, Alice 5700, South Africa; E-Mails: lmabinya@ufh.ac.za (L.V.M.); 200804075@ufh.ac.za (S.C.); 201101482@ufh.ac.za (U.N.)

**Keywords:** *Arthrobacter* sp. Raats, freshwater, bioflocculant, polyssacharide

## Abstract

A bioflocculant-producing bacteria was isolated from Tyume River in the Eastern Cape Province, South Africa and identified by *16S rRNA* gene nucleotide sequence to have 91% similarity to *Arthrobacter* sp. 5J12A, and the nucleotide sequence was deposited in GenBank as *Arthrobacter* sp. Raats (accession number HQ875723). The bacteria produced an extracellular bioflocculant when grown aerobically in a production medium containing glucose as sole carbon source and had an initial pH of 7.0. Influences of carbon, nitrogen and metal ions sources, as well as initial pH on flocculating activity were investigated. The bacteria optimally produced the bioflocullant when lactose and urea were used as sole sources of carbon and nitrogen respectively with flocculating activities of 75.4% and 83.4% respectively. Also, the bacteria produced the bioflocculant optimally when initial pH of the medium was 7.0 (flocculating activity 84%), and when Mg^2+^ was used as cation (flocculating activity 77%). Composition analyses indicated the bioflocculant to be principally a glycoprotein made up of about 56% protein and 25% total carbohydrate.

## 1. Introduction

Presently, both organic and inorganic flocculating agents are extensively used for sedimentation of colloidal and cellular materials and thus applied in a wide range of industrial fields, such as purification of drinking water, wastewater treatment, food industries, dredging and fermentation processes [1]. Notwithstanding their harmfulness to humans and the environment, various chemically synthesized flocculants such as aluminum salts and polyacrylamide derivatives are commonly employed in these processes due to cost considerations and their effectiveness [1,2]. In order to circumvent the health and environmental problems attributed to inorganic and synthetic flocculants, flocculants produced by microorganisms have attracted considerable scientific and technological attention in recent years [3].

The industrial potential of bioflocculants has long been recognized because of their harmlessness, biodegradability and lack of secondary pollution from their degradative intermediates [4]. The majority of bioflocculants produced by different microorganisms are usually high molecular weight polymers such as polysaccharides, proteins, glycoproteins and nucleic acids [5,6]. Although a number of microorganisms have been screened for their bioflocculant-producing capabilities, to-date, very little has been accomplished on a commercial scale [1]. The high cost of production coupled with low yield seem to be the major deterring factors in the advancement of research in developing bioflocculants for both scientific and commercial applications [7]. However, in order to reduce costs and optimize cultivation conditions, strategies such as fed-batch production processes are being developed [8].

As part of our exploration for new and novel bioflocculants that could stand as alternatives to inorganic and synthetic flocculants, the present study reports on the bioflocculant-producing potential of a freshwater bacteria identified as *Arthrobacter* sp. Raats which was isolated from Tyume River, South Africa.

*Arthrobacter* is a common genus of soil bacteria with all species being generally non-sporulating, Gram-positive and obligate aerobes that exhibit pure respiratory metabolism with the exception of at least two species, *Arthrobacter globiforms* and *Arthrobacter nicotianae* which exhibit anaerobic metabolism [9]. Due to their metabolic diversity, *Arthrobacter* species have been used in industrial applications in the bioremediation of contaminated water [9]. The usefulness of *Arthrobacter globiformis* for bioremediation of industrial wastes rich in nitrates was demonstrated by its ability to remove all nitrate from solutions containing from 100 to 150 mM NO_3_^−^ [10], while Shen *et al*. [11] reported on an *Arthrobacter nitroguajacolicus* strain with the capability to transform acrylonitrile into acrylic acid. Su *et al*. [12] also studied the flocculability of the *Arthrobacter* sp. and demonstrated *Arthrobacter albidus* LC13^T^ to be an effective flocculant-producing strain with high flocculating efficiency obtained for the produced bioflocculant LC13-SF.

## 2. Results and Discussion

### 2.1. Identity of the Bacteria

**Figure 1:**
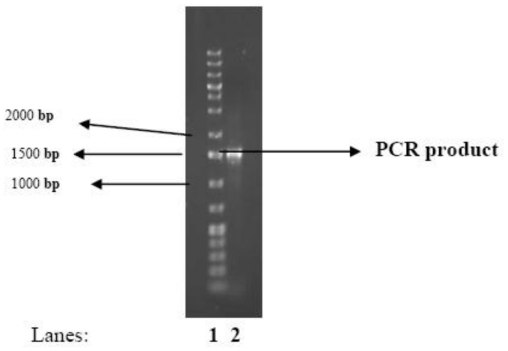
Agarose gel electrophoresis of PCR product of *Arthrobacter* sp. Raats. Lanes: 1 = Ladder; 2 = PCR product.

The test bacterium was one of several isolated from Tyume River in the Eastern Cape Province of South Africa that was screened for bioflocculant-production potential. Initial screening revealed the bacteria to be capable of flocculating kaolin clay suspension (4 g/L) at pH 7.0. Polymerase chain reaction (PCR) amplification of the *16S rRNA* gene of the bacteria produced an amplicon of expected size (approx. 1.5 kb) (Figure 1). The *16S rRNA* gene nucleotide sequence analyses revealed a 91% similarity of the test bacterium to *Arthrobacter* sp. 5J12A, and the nucleotide sequences were deposited in GenBank as *Arthrobacter* sp. Raats with accession number HQ875723.

### 2.2. Effect of Culture Conditions on Bioflocculant Production

Factors such as the constituents of the production medium and culture conditions have an influence on bioflocculant production [13]. Different key factors such as carbon source, nitrogen source, metal ions and the pH were evaluated for their effects on the flocculating activity of the bioflocculant produced by the test bacteria. The effects of lactose, sucrose, fructose and starch on flocculation were assessed and the results are as shown in Table 1. Lactose and sucrose were favorable carbon sources with flocculating activities of 75.4% and 73.4% being attained respectively, while fructose and starch did not support any flocculating activities (Table 1). With respect to sucrose, these results seem to support the findings reported by Li *et al*. [3] for bioflocculant production by *Bacillus licheniformis* X14. However, contrary to the present study, starch was found to enhance the production of the bioflocculant by *Bacillus licheniformis* X14 while lactose was not favored at all [3]. Studies carried out by Patil *et al*. [14] and He *et al*. [15] on bioflocculant production by *Azotobacter indicus* and *Halomonas* sp. V3a’ respectively, also showed a better bioflocculant recovery rate with sucrose as a carbon source. Starch has also been reported as a poor carbon source for bioflocculant production by *Bacillus* sp. Gilbert [16] and *Virgibacillus* sp. Rob [17] respectively with fructose also shown not to be a favorable carbon source among sugars tested for bioflocculant production by *Enterobacter* sp. BY-29 [18].

The effect of nitrogen sources on biofloccdulant production was investigated by cultivating the strain in the same medium, except that the nitrogen source was varied (Table 1). *Arthrobacter* sp. Raats was able to utilize both organic (urea) and inorganic (ammonium sulphate) nitrogen sources with urea being more effectively used and resulting in the highest flocculating activity of 83.4%, followed by ammonium sulphate at 79% and ammonium chloride at 61% (Table 1). Patil *et al*. [14] assessed the influence of nitrogen sources on bioflocculant production by *Azotobacter indicus* and showed that in addition to yeast extract, both urea and ammonium sulphate were also effective in promoting flocculation. On the other hand, ammonium chloride has been reported to be an effective nitrogen source for bioflocculant production by *Halomonas* sp. V3a’ and AAD6 respectively [15,19]. Nevertheless, contrary to the present studies with *Arthrobacter* sp. Raats, both yeast extract and peptone were found to be key parameters influencing optimal production of the bioflocculant produced by *Arthrobacter albidus* LC13^T^ [12]. A number of strains have also been reported to optimally produce bioflocculants in the presence of organic nitrogen sources (or sometimes combined organic and inorganic sources) [20,21].

The production of bioflocculant is influenced by the presence of cations in the culture medium [1]. The effects of various cations on flocculating activity were evaluated using solutions of FeCl_3_, MgCl_2_, FeSO_4_·7H_2_O and KCl as sources of cations. As can be seen in Table 1, the addition of a divalent cation, Mg^2+^ enhanced flocculation when compared to the monovalent cation, K^+^. Both Fe^3+^ and Fe^2+^ had no effect on flocculation. Increased flocculation in the presence of Mg^2+^ was also reported for *Virgibacillus* sp. Rob [17]. In contrast, for bioflocculant LC13-SF produced by *Arthrobacter albidus* LC13^T^, maximum flocculating efficiency required the presence of Ca^2+^ [12] A similar requirement was also reported for optimal flocculation by bioflocculant-producing *Vagococcus* sp. W31 and *Halomonas* sp. V3a’ [22,23]. For *Serratia ficaria*-produced bioflocculant, addition of both divalent cations, Ca^2+^ and Mg^2+^ enhanced the flocculating activity whereas the co-presence of trivalent cations Al^3+^ and Fe^3+^ negatively affected the flocculating activity [20]. Li *et al*. [24] reported a requirement for the co-presence of both monovalent and divalent cations for enhancing flocculating efficiency by *Aeromonas* sp.

Cations were often added to achieve high flocculating activity by neutralizing negatively charged functional groups of both the bioflocculant molecules and the suspended particles [1,25] and consequently weaken the static repulsive force thus enhancing the flocculating effect [2]. The influence of these cations on flocculating rates varies among bioflocculant-producing microorganisms.

**Figure 2:**
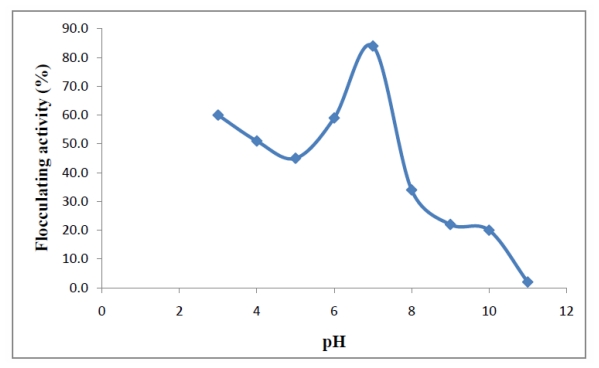
Effect of initial pH on flocculating activity.

The pH of the solution is also a key factor in flocculation and thus effectively influences the flocculation process [26]. The effect of initial pH of the medium on flocculating activity by *Arthrobacter* sp. Raats was investigated at pH values ranging from 3 to 11, and the flocculating activity was found to be distinctly higher (84%) in neutral pH conditions (pH 7.0) than in acidic and alkaline conditions (Figure 2). Similar pH values (pH 7.2 and 7.0) were reported for optimum activity of bioflocculants ERSS-31 produced by *Halomonas maura* sp. nov. [27] and MBFW31 produced by *Vagococcus* sp. W31 [22] respectively. Bioflocculant HBF-3, produced by a mutant *Halomonas* sp. V3a’ also attained the highest flocculating activity at pH 7 [23]. At low pH, the absorption of H^+^ ions tends to weaken the bioflocculant-kaolin complex formation process and a similar effect is also observed at high pH values due to OH^−^ ions [23]. According to Li *et al*. [25], the mediating effect of cations appears to be strongest at neutral pH values. However, these observations are different from studies carried out by Choi *et al*. [28] and Zheng *et al*. [2] in which the maximum flocculating activities of bioflocculants produced by *Anabaena* sp. and *Bacillus* sp. F19 respectively were observed at pH 2. The bioflocculant produced by *Bacillus* sp. PY-90 was found to be actively high in an acidic pH range of 3.0 to 5.0 [29], while *Serratia ficaria* produced a bioflocculant effective over a weakly acidic pH range of 5.0 to 7.0 [20].

### 2.3. Time Course Assay of Bioflocculant Production

**Figure 3:**
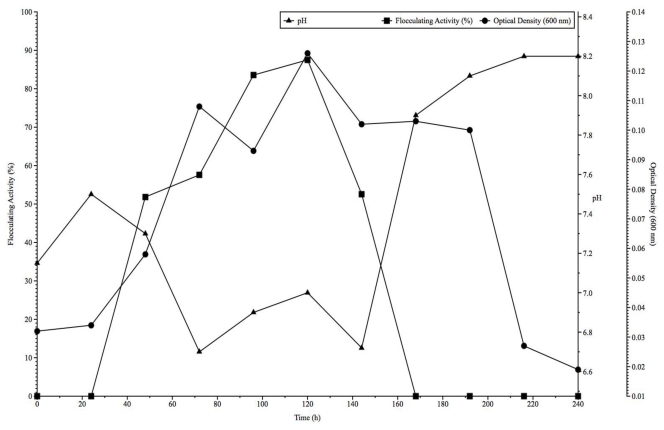
Time course assay of flocculant production by *Arthrobacter* sp. Raats.

Figure 3 shows the time course of bioflocculant production by *Arthrobacter* sp. Raats in relation to optical density, pH and flocculating activity over a 10 days cultivation period. During growth, the flocculating activity was observed to increase reaching a maximum of 87.5% in 5 days beyond which a rapid decline was noted with a complete loss of activity over the last 4 days of growth (Figure 3). The observed decrease in flocculating activity might be due to the activity of deflocculation enzymes in the late phase of cell growth [20,28]. According to a study by Jang *et al*. [30] on bioflocculant production by fed-batch culture of *Citrobacter* sp., specific flocculating activity and bioflocculant production was influenced by fermentation conditions which included among other things, carbon source, inoculum size, dissolved oxygen tension (DOT) and carbon/nitrogen (C/N) source ratio. Jang *et al*. [30] also noted that the initial growth rate increased with an increase in carbon source concentration. However, carbon source consumption was severely limited at higher carbon source concentrations. Inoculum size has also been reported to affect not only cell growth but also product formation with the dynamic behavior of cells of *Citrobacter* sp. cell cultures at different inoculum sizes affecting bioflocculant activity [30,31]. The synthesis of bioflocculant and the flocculating activity were also shown to be significantly affected by the C/N ratio of the growth medium with a higher C/N ratio not necessarily translating to a higher yield of flocculant synthesis [30]. The influence of the initial pH of fermentation medium can determine both the electric charge of the cells and the oxidation-reduction potential which, in turn, can affect nutrient absorption and enzymatic reaction [12]. Thus the corresponding steady increase in cell growth (OD_600_) in relation to flocculating activity observed over the first 5 day growth period is possibly an indication that the bioflocculant was produced by biosynthesis during growth of the bacterium and not by cell autolysis [22]. From 72 to 120 h, the pH increased steadily until optimal flocculating activity was reached at pH 7.0. After 6 days of cultivation, a complete loss of flocculating activity was observed while the pH increased sharply and remained around pH 8.2 (Figure 3). The correlation between bioflocculant production and culturing times may differ among different organisms. Maximum production levels and a flocculating peak of 90.6% for a bioflocculant produced by *Arthrobacter albidus* LC13^T^ were obtained at 72 h of fermentation time under neutral conditions (pH 7.0) [12]. For *Bacillus licheniforms*, maximum values were obtained for both the flocculant production and cell growth during the stationary phase [32]. Li *et al*. [3] and Xia *et al*. [21] showed that for *Bacillus licheniforms* X14, and *Proteus mirabilis* TJ-1, maximum production of the bioflocculant was achieved in less time than required for other strains (24 h) while the flocculating activity peaked during the early stationary phase (48 h). The observed fluctuations in the pH of the medium during bacterial growth may also be attributed, according to Lors *et al*. [33], to two opposite phenomena: a decrease of pH due to the bacterial activity and an increase of pH due to the release of hydroxide ions associated to the cations that are leached out. Taken together, these observations seem to strongly support a fed-batch culture approach in order to fully understand the dynamics involved in a growing cell culture. The relationship between growth kinetics and the concentration of a substrate is fundamental in studying the enormous inconsistencies that exist especially for pure cultures growing with single substrates. Confounding the problem further is the fact that microorganisms tend to change their kinetic properties in order to adapt to changing environment [34].

### 2.4. Chemical Analysis of Bioflocculant Composition

An aliquoted suspension (1 mg/mL) of the purified bioflocculant was found to contain 0.25 mg/mL total carbohydrate and 0.56 mg/mL protein respectively, thus confirming that the bioflucculant consisted primarily of a glycoprotein. Inoue *et al*. [35] investigated a biopolymer produced by *Arthrobacter* sp. and revealed it to be a heterogenous polysaccharide which was principally a galactan sulfate. A number of other organisms have been reported to produce different kinds of glycoprotein bioflocculants [5,36].

## 3. Experimental Section

### 3.1. Test Bacterium and Cultivation Conditions

The test bacterium was one of the bioflocculant producing bacteria isolated from Tyume River in the Eastern Cape Province of South Africa that forms part of the culture collections of the Applied and Environmental Microbiology Research Group (AEMREG), University of Fort Hare, Alice, South Africa. The bacterium was preserved on agar slants and the glycerol (20%) stocks maintained at −80 °C. The composition of the cultivation medium was prepared by mixing the following: glucose (10 g), MgSO_4_·7H_2_O (0.3 g), K_2_HPO_4_ (5 g), peptone (1.0 g) and KH_2_PO_4_ (0.2 g) in a litre of deionized water at pH 7.0 [15,16,36]. A loopful of colonies of the test bacterium was inoculated into 5 mL of the growth medium and incubated in a rotary shaker (160 rpm) at 28 °C for 5 days. The resultant culture broth (2 mL) was centrifuged (8000× g, 15 min) and the cell-free supernatant was assayed for flocculating activity.

### 3.2. Determination of Flocculating Activity

Using a suspension of kaolin clay as test material, flocculating activity was determined according to Kurane *et al*. [37] as modified by Gao *et al*. [22]. A suspension of kaolin clay (4 g/L) in deionized water at pH 7 was used as a stock solution for the subsequent assays. The following solutions were mixed in a test tube: kaolin clay suspension (9 mL), culture supernatant (0.1 mL) and 1% CaCl_2_ (0.25 mL). A control in which the culture supernatant was replaced with deionized water was also included and measured under similar conditions. The final volume of all mixtures was made up to 10 mL with deionized water. The solutions were mixed gently and allowed to settle for 5 min. at room temperature. The optical density (OD) of the clarifying upper phase solution was measured at 550 nm using a ThermoSpectronic spectrophotometer (Helios Epsilon, USA) and the flocculating activity determined as follows:

Flocculating activity=[(B-A)/B]×100%

where *A* and *B* are optical densities at 550 nm of the sample and control respectively.

### 3.3. Effect of Carbon and Nitrogen Sources on Bioflocculant Production

The effects of the following carbon sources were assessed: lactose, sucrose, fructose and starch. Nitrogen source candidates included ammonium sulphate, ammonium chloride and urea. The assessments were done following the method described by Lachhwani [38].

### 3.4. Effect of Various Cations and pH on Bioflocculant Production

The effect of metal ions was evaluated utilizing the procedure elaborated above, but the CaCl_2_ solution was replaced by various salt solutions and the flocculating activity determined. Solutions of KCl, MgCl_2_, FeCl_3_ and FeSO_4_·7H_2_O were used as cation sources. To assess the effect of pH on flocculating activity, the initial pH of the kaolin suspension was varied between the pH range of 3–11 by adjusting with either HCl or NaOH [39].

### 3.5. Time Course Assay of Bioflocculant Production

The composition of the medium for bioflocculant production was prepared according to the method described under subsection 3.1 [36]. The selected strain was pre-cultured in 50 mL medium contained in 50 mL flasks on a rotary shaker (160 rpm) at 28 °C for 16 h and used as seed culture for batch fermentation. Batch fermentations were carried out according to the modified method of Gao *et al*. [22]. Seed culture (10% v/v) was used to inoculate 150 mL of medium in 500 mL flasks on a rotary shaker (160 rpm) at 28 °C. Culture samples were withdrawn at appropriate time intervals (24 h) for a period of 240 h and monitored for growth (OD_660_), pH and flocculating activity. Culture broth (5 mL) was centrifuged (8000× g, 15 min.) and the cell-free supernatant was used to determine the flocculating activity as previously described.

### 3.6. Extraction, Purification and Characterization of the Bioflocculant

After 5 days of fermentation, the culture broth was centrifuged (8000× g, 15 min.) to remove bacterial cells. One volume of distilled water was added to the supernatant phase and centrifuged (8000× g, 15 min.) to remove insoluble substances. The supernatant was then mixed with 2 volumes of ethanol, stirred and left standing at 4 °C for 12 h. The supernatant was decanted and the precipitate vacuum-dried to obtain the crude biopolymer. The crude product was then dissolved in distilled water and mixed with 1 volume of chloroform/*n*-butyl alcohol (5:2, v/v). After stirring, the mixture was left standing at room temperature for 12 h. The upper phase was separated, centrifuged (3000× g, 15 min.) and the supernatant concentrated at 40 °C. Two volumes of ethanol were added, the precipitate recovered, vacuum-dried and re-dissolved in distilled water. Thereafter, the protein content was measured using the Folin-Lowry method, and total carbohydrate was assayed using Phenol-Sulphuric acid protocol as described by Lachhwani [38].

### 3.7. Identification of the Bioflocculant-Producing Microorganism

#### DNA extraction

DNA extraction was conducted via the boiling method whereby 2–3 colonies were suspended in 70 μL of sterile double distilled water. The samples were heated in a water bath at 100 °C for 10 min, allowed to cool for 5 min and thereafter centrifuged at 3000 rpm for 5 min. The supernatant was transferred to a clean tube and stored at 4 °C. This served as the template in the PCR assay.

#### PCR Amplification

PCR was carried out in 50 μL reaction volume containing 2 mM MgCl_2_, 2 U Supertherm Taq polymerase, 150 mM of each dNTP, 0.5 mM of each primer (F1: 59-AGAGTTTGATCITGGCTCAG-39; I = inosine and primer R5: 59-ACGGITACCTTGTTAC GACTT-39) and 2 μL template DNA. Primer F1 and R5 binds to base positions 7–26 and 1496–1476 of the *16S rRNA* gene of *Streptomyces ambofaciens* ATCC 23877, respectively [40]. The primers in this study were used to amplify nearly full-length 16S rDNA sequences. The PCR programme used was an initial denaturation (96 °C for 2 min), 30 cycles of denaturation (96 °C for 45 s), annealing (56 °C for 30 s) and extension (72 °C for 2 min), and a final extension (72 °C for 5 min). Gel electrophoresis of PCR products were conducted on 1% agarose gels to confirm that a fragment of the correct size had been amplified.

Automated sequencing of the *16S rRNA* genes of the bacterial isolates was performed using the Spectrumedix SCE2410 genetic analysis system with 24 capillaries. The sequencing reactions were performed according to the manufacturer’s instructions, using the Big Dye version 3.1 dye terminator cycle sequencing kit (Applied Biosystems) and 27F primer. The sequences were edited manually based on the most similar sequences.

## 4. Conclusions

The bioflocculant produced by *Arthrobacter* sp. Raats showed good flocculating activity for kaolin suspension. Lactose and urea were preferred as sole carbon and nitrogen sources for bioflocculant production by the bacteria. The divalent cation (Mg^2+^) as well as initial medium pH 7.0 resulted in optimal production of bioflocculant, and chemical analyses indicated the bioflocculant to be a glycoprotein made up of about 56% protein and 25% total carbohydrate. *Arthrobacter* sp. Raats appears to hold promise as a source of new bioflocculant that could stand as an alternative to inorganic and synthetic organic flocculants.

## Figures and Tables

**Table 1:** Effect of constituents of culture medium on the production and flocculating activity of the bioflocculant produced by *Arthrobacter* sp. Raats.

**Carbon Source**	**Lactose**	**Sucrose**	**Fructose**	**Starch**
Flocculating Activity %	75.4	73.4	2.8	2.1

**Nitrogen Source**	**Ammonium Chloride**	**Ammonium Sulphate**	**Urea**	
Flocculating Activity %	60.9	78.5	83.4	

**Cations**	**Ferric Chloride**	**Magnesium Chloride**	**Ferrous Sulphate**	**Potassium Chloride**
Flocculating Activity %	-	76.9	-	2.8

Note: - denotes no flocculating activity detected.

## References

[1] Salehizadeh H., Shojaosadati S.A. (2001). Extracellular biopolymeric flocculants: Recent trends and biotechnology importance. Biotechnol. Adv.

[2] Zheng Y., Ye Z.-L., Fang X.-L., Li Y.-H., Cai W.-M. (2008). Production and characteristics of a bioflocculant produced by *Bacillus* sp. F19. Bioresour. Technol.

[3] Li Z., Zhong S., Lei H.-Y., Chen R.-W., Yu Q., Li H.-L. (2009). Production of a novel bioflocculant by *Bacillus licheniformis* X14 and its application to low temperature drinking water treatment. Bioresour. Technol.

[4] Wang L., Ma F., Qu Y., Sun D., Li A., Guo J., Yu B. (2011). Characterization of a compound bioflocculant produced by mixed culture of *Rhizobium radiobacter* F2 and *Bacillus sphaeicus* F6. World J. Microbiol. Biotechnol.

[5] Feng D.L., Xu S.H. (2008). Characterization of biofloculant MBF3-3 produced by an isolated *Bacillus* sp. World J. Microbiol. Biotechnol.

[6] Wu J., Ye H.-F. (2007). Characterization and flocculating properties of an extracellular biopolymer produced from a *Bacillus subtillis* DYU1 isolate. Process Biochem.

[7] Li Y., He N., Guan H., Du G., Chen J. (2003). A novel polygalacturonic acid bioflocculant REA-11 produced by *Corynebacterium glutamicum*: A proposed biosynthetic pathway and experimental confirmation. Appl. Microbiol. Biotechnol.

[8] Wu H., Li Q., Lu R., Wang Y., Zhuang X., He N. (2010). Fed-batch production of a bioflocculant from *Corynebacterium glutamicum*. J. Ind. Microbiol. Biotechnol.

[9] Eschbach M., Möbitz H., Rompf A., Jahn D. (2003). Members of the genus Arthrobacter grow anaerobically using nitrate ammonification and fermentative processes: Anaerobic adaptation of aerobic bacteria abundant in soil. FEMS Microbiol. Lett.

[10] Piñar G., Ramos J.L. (1998). A strain of *Arthrobacter* that tolerates high concentrations of nitrate. Biodegradation.

[11] Shen M., Zeng Y.-G., Shen Y.-C. (2009). Isolation and characterization of a novel *Arthrobacter nitroguajacolicus* ZJUTB06-99, capable of converting acrylonitrile to acrylic acid. Process Biochem.

[12] Su X., Shen X., Ding L., Yokota A. (2011). Study on the flocculability of the *Arthrobacter* sp., an actinomycete resuscitated from the VBNC state. World J. Microbiol. Biotechnol.

[13] He N., Li Y., Chen J. (2004). Production of a polygalacturonic acid bioflocculant REA-11 by *Corynebacterium glutamicum*. Bioresour. Technol.

[14] Patil S.V., Salunkhe R.B., Patil C.D., Patil D.M., Salunke B.K. (2010). Bioflocculant exopolysaccharide production by *Azotobacter indicus* using flower extract of *Madhuca latifolia* L. Appl. Biochem. Biotechnol.

[15] He J., Zhen Q., Qiu N., Liu Z., Wang B., Shao Z., Yu Z. (2009). Medium optimization for the production of a novel bioflocculant from Halomonas sp. V3a’ using response surface methodology. Bioresour. Technol.

[16] Piyo N., Cosa S., Mabinya V.L., Okoh I.A. (2011). Assessment of bioflocculant production by *Bacillus* sp. Gilbert, a marine bacterium isolated from the bottom sediment of Algoa Bay. Mar. Drugs.

[17] Cosa S., Mabinya L.V., Olaniran A.O., Okoh O.O., Bernard K., Deyzel S., Okoh A.I. (2011). Bioflocculant production by *Virgibacillus* sp. Rob isolated from the bottom sediment of Algoa Bay in the Eastern Cape, South Africa. Molecules.

[18] Yokoi H., Hirose J., Hayashi S., Takasaki Y. (2001). Simultaneous production of hydrogen and bioflocculant by *Enterobacter* sp. BY-29. World J. Microbiol. Biotechnol.

[19] Sam S., Kucukasik F., Yenigun O., Nicolaus B., Oner E.B., Yukselen M.A. (2011). Flocculating performances of exopolysaccharides produced by a halophilic bacterial strain cultivated on agro-industrial waste. Bioresour. Technol.

[20] Gong W.-X., Wang S.-G., Sun X.-F., Liu X.-W., Yue Q.-Y., Gao B.-Y. (2008). Bioflocculant production by culture of *Serratia ficaria* and its application in wastewater treatment. Bioresour. Technol.

[21] Xia S., Zhang Z., Wang X., Yang A., Chen L., Zhao J., Leonard D., Jaffrezic-Renault N. (2008). Production and characterization of bioflocculant by *Proteus mirabilis* TJ-1. Bioresour. Technol.

[22] Gao J., Bao H.-Y., Xin M.-X., Liu Y.-X., Li Q., Zhang Y.-F. (2006). Characterization of a bioflocculant from a newly isolated *Vagococcus* sp. W31. J. Zhejiang Univ. Sci. B.

[23] He J., Zou J., Shao Z., Zhang J., Liu Z., Yu Z. (2010). Characteristics and flocculating mechanism of a novel bioflocculant HBF-3 produced by deep-sea bacterium mutant *Halomonas* sp. V3a’. World J. Microbiol. Biotechnol.

[24] Li X.-M., Yang Q., Huang K., Zeng G.-M., Liao D.-X., Liu J.-J., Long W.-F. (2007). Screening and characterization of a bioflocculant produced by *Aeromonas* sp. Biomed. Environ. Sci.

[25] Li W.W., Zhou W.Z., Zhang W.Z., Wang J., Zhu X.B. (2008). Flocculation behaviour and mechanism of an exopolysaccharide from the deep-sea psychrophilic bacterium *Pseudoalteromonas* sp. SM9913. Bioresour. Technol.

[26] Yokoi H., Arima T., Hirose J., Hayashi S., Takasaki Y. (1996). Flocculation properties of poly (γ-glutamic acid) produced by *Bacillus subtilis*. J. Ferment. Bioeng.

[27] Bouchotroch S., Quesada E., Del Moral A., Llamas I., Béjar V. (2001). *Halomonas maura* sp. nov., a novel moderately halophilic, exopolysaccharide-producing bacterium. Int. J. Syst. Evol. Microbiol.

[28] Choi C.W., Yoo S.-A., Oh I.-H., Park S.H. (1998). Characterization of an extracellular flocculating substance produced by a planktonic cyanobacterium, *Anabaena* sp. Biotechnol. Lett.

[29] Yokoi H., Natsuda O., Hirose J., Hayashi S., Takasaki Y. (1995). Characteristics of a biopolymer flocculant produced by *Bacillus* sp. PY-90. J. Ferment. Bioeng.

[30] Jang J.-H., Ike M., Kim S.M., Fujita M. (2001). Production of a novel bioflocculant by fed-batch culture of *Citrobacter* sp. Biotechnol. Lett.

[31] Kallos M.S., Behie L.A. (1999). Inoculation and growth conditions for high-cell-density expansion of mammalian neural stem cells in suspension bioreactors. Biotechnol. Bioeng.

[32] Shih I.L., Van Y.T., Yeh L.C., Lin H.G., Chang Y.N. (2001). Production of a biopolymer flocculant from *Bacillus licheniformis* and its flocculation properties. Bioresour. Technol.

[33] Lors C., Chehade M.H., Damidot D. (2009). pH variations during growth of *Acidithiobacillus thiooxidans* in buffered media designed for an assay to evaluate concrete biodeterioration. Int. Biodeterior. Biodegrad.

[34] Kovárová-Kovar K., Egli T. (1998). Growth kinetics of suspended microbial cells: From single-substrate-controlled growth, to mixed-substrate kinetics. Microbiol. Mol. Biol. Rev.

[35] Inoue K., Korenaga H., Kadoya S. (1982). A sulfated polysaccharide produced by an *Arthrobacter* species. J. Biochem.

[36] Zhang Z.-Q., Lin B., Xia S.-Q., Wang X.-J., Yang A.-M. (2007). Production and application of a novel bioflocculant by multiple microorganism consortia using brewery wastewater as carbon source. J. Environ. Sci.

[37] Kurane R., Takeda K., Suzuki T. (1986). Screening and characteristics of microbial flocculants. Agric. Biol. Chem.

[38] Lachhwani P. (2005). Studies on Polymeric Bioflocculant Producing Microorganisms. Master Thesis.

[39] Yim J.H., Kim S.J., Ahn S.H., Lee H.K. (2006). Characterization of novel bioflocculant, p-KG03, from a marine dinoflagellate, *Gyrodiumimpudicum* KG03. Bioresour. Technol.

[40] Cook A.E., Meyers P.R. (2003). Rapid identification of filamentous actinomycetes to the genus level using genus-specific *16 S rRNA* gene restriction fragment patterns. Int. J. Syst. Evol. Microbiol.

